# Reusable 3D-Printed Thermoplastic Polyurethane Honeycombs for Mechanical Energy Absorption

**DOI:** 10.3390/polym17223035

**Published:** 2025-11-16

**Authors:** Alin Bustihan, Razvan Hirian, Ioan Botiz

**Affiliations:** 1 Department of Physics of Condensed Matter and Advanced Technologies, Faculty of Physics, Babeș-Bolyai University, 400084 Cluj-Napoca, Romania; alin.bustihan@stud.ubbcluj.ro (A.B.); razvan.hirian@ubbcluj.ro (R.H.); 2 Interdisciplinary Research Institute on Bio-Nano-Sciences, Babeș-Bolyai University, 400271 Cluj-Napoca, Romania

**Keywords:** thermoplastic polymers, 3D printing, honeycomb structures, mechanical properties, impact protection applications

## Abstract

In this study, we investigate the mechanical energy absorption performance of reusable 3D-printed honeycomb structures fabricated using fused deposition modeling with three thermoplastic polyurethane variants: TPU 70A, TPU 85A, and TPU 95A. Prior to manufacturing, the mechanical properties of the TPU filaments were analyzed as a function of printing temperature to optimize tensile strength and layer adhesion. Four honeycomb configurations, including hexagonal and circular cell geometries, both with and without a 30° twist, were subjected to out-of-plane compression testing to evaluate energy absorption efficiency, specific energy absorption, and crushing load efficiency. The highest energy absorption efficiency, 47%, was achieved by the hexagonal honeycomb structure fabricated from TPU 95A, surpassing the expected values for expanded polystyrene and approaching the performance reported for high-cost advanced lattice structures. Additionally, twisted honeycomb configurations exhibited improved crushing load efficiency values (up to 73.5%), indicating better stress distribution and enhanced reusability. Despite variations in absorbed energy, TPU 95A demonstrated the best balance of elasticity, structural integrity, and reusability across multiple compression cycles. These findings suggest that TPU-based honeycomb structures could provide a viable, cost-effective alternative for energy-absorbing applications in impact protection systems, automotive safety, and sports equipment.

## 1. Introduction

Polymers are a class of soft materials [1,2] widely used to fabricate miniaturized surface relief structures and nanoscale features through both top-down and bottom-up approaches [3,4,5,6,7]. Their applications span various fields of materials science, including medicine, electronics, energy, and more. The emergence of additive manufacturing techniques for polymers has further diversified polymeric structures by enabling the construction of three-dimensional (3D) objects from digital models via layer-by-layer deposition, sintering, or curing. This advancement has significantly expanded the applicability of polymers to additional industrial sectors such as prototyping, tooling, and safety applications.

One of the most fascinating nature-inspired macroscopic configurations capable of efficiently absorbing mechanical energy is the honeycomb structure [8,9,10]. This key capability is primarily attributed to the structure’s periodic cellular design, which provides a superior strength-to-weight ratio, controlled deformation, and effective load distribution under impact [11]. The honeycomb structure features a repeating pattern of hexagonal cells, allowing for progressive and stable deformation during compression, making it an optimal choice for applications where energy dissipation is critical [12]. Moreover, mechanical energy resulting from compression can be absorbed through various mechanisms (e.g., plastic or elastic deformation, buckling). The crushing behavior of honeycomb structure can be divided into three stages: elastic deformation, progressive collapse, and densification [13]. This complex deformation mechanism ensures progressive absorption of high energy [14]. Consequently, the mechanical properties of honeycomb structures are influenced by several factors, including the type of material used and the manufacturing method [15,16].

For example, while metallic structures achieve optimal mechanical energy absorption through complete plastic deformation, composite materials [17] and polymer-based structures absorb energy by progressively compressing the mechanical energy absorption structure (MEAS) [17,18,19]. Both absorption methods typically result in the complete destruction of the structure, allowing only limited reusability of the MEAS. Nevertheless, qualitative studies have been conducted on single-use mechanical energy absorption structures inspired by nature, including reproductions of wood structures and turtle shell re-entrant arc-shaped structures [20]. Although turtle shells exhibit dynamic response curves with better crushing load uniformity than conventional re-entrant honeycombs, wood structures inspired by balsa [21], bamboo [22], or cork [23] generally demonstrate superior energy absorption capabilities due to their fundamentally different internal architectures based on 2D honeycomb configurations. Specifically, the balsa wood structure typically consists of second-order, wood-inspired hierarchical honeycombs, where the solid side of a conventional honeycomb is replaced by a wall composed of smaller hexagons that absorb mechanical energy in the in-plane direction [21]. In contrast, the bamboo configuration features bio-inspired hierarchical circular thin-walled honeycomb structures designed to efficiently absorb energy under out-of-plane crushing loads [22]. In comparison, cork structures exhibit a chambered architecture that closely resembles the so-called 3D triply periodic minimal surface structures (TPMS) which are defined as highly porous geometries that repeat periodically in three dimensions while maintaining a minimal surface area for a given volume. Cork structures absorb impact isotropically, meaning they absorb approximately the same amount of energy regardless of the direction of the force (whether in-plane or out-of-plane).

Other structures designed to absorb mechanical energy have been reported in the literature, including those that imitate Miura origami-type folded shapes [24], “kagome lattice” structures inspired by traditional Japanese woven patterns [25], and flexible fish scale-like configurations [26]. While each of these materials offers distinct advantages, they all share the drawback of limited or no reusability. Miura origami-type folded shapes are efficient mechanical energy absorbers due to their ability to collapse in a controlled manner, absorbing energy through plastic deformation. In contrast, “kagome lattice” structures exhibit superior energy dissipation properties because of their truss arrangement, which enables efficient load distribution, progressive buckling, and enhanced resistance to shear and impact forces. Their combination of axial stretching and bending mechanisms allows for gradual energy absorption, reducing stress concentrations and preventing sudden structural failure [25]. Fish scale-like structures provide good impact resistance while maintaining flexibility, a characteristic attributed to their overlapping hierarchical arrangement [26].

Furthermore, it is worth mentioning that all the aforementioned structures are considered effective mechanical energy absorbers based on several physical parameters, including specific energy absorption (SEA). Generally, a material becomes a better energy absorber as its SEA increases, as demonstrated, for example, by bio-inspired honeycomb structures made from bamboo or pomelo peel [27]. Moreover, the SEA parameter becomes even more critical when designing and manufacturing high-capacity mechanical energy absorption systems (MEAS) that are extremely lightweight and used in applications such as safety helmets [28], automotive passive safety, highway safety, packaging of valuables [29,30], and the aerospace industry [31].

In this work, we propose a series of innovative and reusable honeycomb structures fabricated from three types of thermoplastic polyurethane (TPU) filaments using a fused deposition modeling (FDM) 3D printing. Although various polymeric materials have been used in previous studies dedicated to the fabrication of MEAS, including polylactic acid [32,33,34], Nylon [35,36], and acrylonitrile butadiene styrene [34,37], TPU has been recognized for its elasticity and processability [31,38,39]. However, the potential of TPU for creating highly reusable, high-efficiency honeycombs through systematic FDM optimization remains underexplored. TPU combines a favorable mix of flexibility, resilience and low cost, and its tunable hardness and good printability make it especially attractive for printed MEAS that must recover after impact [12,39]. Crucially, the mechanical response of filament-assembled TPU architectures is dominated by process-dependent interlayer adhesion and printing parameters; therefore, producing reusable TPU honeycombs requires deliberate parameter optimization rather than simple material substitution. In this study, we address this gap by comparing multiple TPU grades, mapping key FDM parameters to mechanical performance, and demonstrating reusability through repeated out-of-plane compression tests supported by before-and-after inspections. Compared with single-use, plastically deforming foams and more costly powder- or beam-based additive manufacturing methods (SLS/SLM, PolyJet, EBM) [40,41,42,43], optimized FDM of TPU offers a low-cost, rapid, and lightweight approach to scalable, reusable MEAs with reduced material waste and improved suitability for safety and automotive applications [12,29,31,38,39,40,41,42,43,44]. Thus, our specific objectives are to optimize FDM printing parameters for three TPU variants; characterize the out-of-plane compression behavior of four honeycomb geometries (hexagonal and circular, with and without twist); evaluate key performance metrics including energy absorption efficiency, specific energy absorption (SEA), and crushing load efficiency (CLE); and assess reusability potential across multiple compression cycles through repeated testing and before-and-after inspections. To achieve these goals, we combine systematic process–property mapping with mechanical testing and structural inspection to identify processing windows that maximize both energy absorption performance and recovery.

## 2. Materials and Methods

### 2.1. Utilized Polymers and Their Characteristics

For the printing of all honeycomb structures presented in this work, we have used the following polymer filaments: TPU-95A (from Polymaker PolyFlex, Changshu, Jiangsu Province, China), TPU-85A (from NinjaTek NinjaFlex, Manheim, PA, USA) and TPU-70A (from Recreus FilaFlex, Alicante, Spain). Their corresponding (mechanical) characteristics are listed in Table 1. All filaments are based on TPU [45], a flexible linear segmented copolymer based on a hard polymer segment (HS) and soft polymer segment (SS).

The three types of TPU were used to produce 3D-printed honeycomb MEASs of different hardness, with the final goal of studying their energy absorption capabilities. Moreover, TPU 95A has been chosen as a starting material because it is a widely used material on the market, and thus, there are many sources of information about how to print it using the features of our 3D printer. The other two materials (TPU 85A and TPU 70A) were employed in order to test the impact of resulting MEAS hardness on the energy absorption properties. Note that the mechanical properties of these filaments are not the same as the properties of the final honeycomb 3D-printed structures, as the printing of the honeycomb structures was done layer by layer, with layers’ adhesion property also coming into play (typically, this adhesion depends on a number of physico-chemical parameters, the most important being the printing temperature of the filament). All honeycomb structures were prepared using five different printing temperatures each increasing from 210 °C with an increment of 5 °C.

### 2.2. Printing Parameters and CAD & Slicer

Our MEAS samples were 3D-printed using a Modix BIG60 FDM 3D printer (Tel Aviv, Israel), equipped with a Titan Aero extruder and a 0.4 mm hardened steel Volcano nozzle from E3D (Oxfordshire, UK). This type of printer is based on extrusion printing at a specific temperature of a polymer filament, and thus, it is able to print samples of widely available materials at low costs. Moreover, the bed material was made of polyetherimide (PEI), while the bed temperature was set at 60 °C for all printed MEASs. We further used a water-soluble polyvinyl alcohol (PVA) intermediate layer to favor bonding of printed structures with the bed. The following parameters were used for printing: a 30 mm/s maximum printing speed both for the walls and infill, 100% fan speed while the chamber was open, and a bed temperature of 50 °C for all tensile strength samples. For more uniform printing results, we set the material flow to 120% and the infill of the samples to 30%. For the walls a wall line count of 3 was used (under such conditions, the exterior walls of the tensile strength test samples will have a 1.2 mm thickness, which is equivalent to three times the diameter of the extrusion nozzle hole). Finally, to generate the 3D honeycomb models, we utilized the SolidWorks 2020 application, which is a computer-aided engineering (CAE) software that allows the design and assembly of 3D models. The G-code for 3D printing of our MEAS samples was made using the slicer program Cura 5.3.0.

### 2.3. Samples Modeling

From the most common structures reported in the literature to be efficient for the absorption of mechanical energy [49], we have chosen the four similar models of honeycomb structures, each differing by a number of specific parameters, such as the shape of the base cell or the angle of the base cell. The height of all samples was set to 20 mm, and the outer hexagonal contour of each base cell was selected to be 5.5 mm in the case of basic cells with a hexagonal hole and 5.2 mm in the case of those with a circular hole, respectively. All 3D-printed honeycomb structures comprised 55 basic cells. Schematics depicting a hexagonal hole cell, and a circular hole cell, along with their dimensions, can be observed in Figure S1 in the Supporting Information. Because the shape of these holes dictate the geometric characteristics of the structures, the walls in the hexagonal case had a constant thickness of 0.5 mm. For the circular cell configuration, the wall thickness was designed with a minimum thickness of 0.5 mm, a feature that plays a critical role in the mechanical response. This reduced thickness region acts as a natural stress concentrator, influencing the onset and propagation of local failure under compressive loading. The link between this geometric feature and the resulting stress concentration and collapse behavior is discussed in Section 3, where its impact on the deformation and failure mechanisms is analyzed. Additionally, in order to investigate whether we can obtain tilted TPU honeycomb structures displaying superior results in terms of energy absorbance efficiency or crushing load efficiency (CLE), comparable to structures made from metallic materials (Ti6Al4V) [50], we have also printed honeycomb structures in which the upper plane of the structure is rotated by 30° with respect to the base. The height of these structures was kept the same as for the other samples, i.e., limited to 20 mm. All four finite element models of MEAS are presented in Figures S2 and S3 in the Supporting Information.

### 2.4. Mechanical Tensile Strength and Compression Tests

Tensile strength tests were performed in order to study the maximum force and elongation at which the sample yields. Each test was a standard ISO 527-1:2019 [51] test specially designed for plastics. Each sample, connected between the machine grips, was pulled at a predetermined speed of 10 mm/minute until it failed/broke. All tests were performed on a Mecmesin MultiTest 5-I device (Horsham, UK), while using a standard ASTM-D628-10 [52] Type V breaking tests sample. The specimen was printed vertically, as it can be observed in Figure S4 in the Supporting Information, so that the pulling direction was the same as printing direction. Compression tests consisted in placing a honeycomb MEAS sample between two metal plates, while the lower surface was fixed and the upper one could move at 10 mm/min. All compression tests were carried on the Mecmesin (West Sussex, UK) MultiTest 5-I device.

For the first tensile strength test we have characterized the samples calculating the maximum elongation, maximum breaking force, and Young’s modulus (*E*). The latter was computed in arbitrary units, as at certain temperatures the central area of the sample exhibited slight variations attributed to polymer degradation at higher temperatures. The maximum elongation was calculated as:

(1) $$\varepsilon_{max}=\frac{l_{max}}{l_{0}}$$

Here $l_{max}$ was the maximum length of the standard ASTM-D628-10 Type V sample measured by the test machine, while $l_{0}$ was the starting dimension of the sample between the machine grips.

The breaking force, $F_{max}$ was the maximum force measured by the testing machine, and characterizing the moment a sample would break due to applied stress. In this case, the Young’s modulus, it was calculated as the slope of the force-elongation curve in the force–strain graphical representation.

### 2.5. Mechanical Energy Absorption Parameters

For all MEAS samples, the absorption capacity of the structures was characterized by a series of standard parameters reported in the literature [38] and further described below. Figure S5, presented in the Supporting Information, visually shows how to set parameters such as strain before the densification zone (*ε_D_*), maximum peak strain (*ε*_0_) or specific stress of maximum peak ($\sigma_{v}$).

***Absorbed energy (E_abs_)*** was defined as the area under the compression graph (stress as a function of load) to the densification zone [53], and has the formula:

(2) $$E_{abs}=\int_{0}^{\varepsilon_{D}}\sigma \left(\varepsilon \right)d\varepsilon$$

Here σ represents the stress and ε denotes the strain.

***Specific energy of absorption (SEA)*** was defined as the energy absorbed per unit of mass [54], and denotes the ability of a specific structure to store energy:

(3) $$SEA=\frac{E_{abs}}{m}$$

Here *m* represents the mass of the structure.

***Specific stress of the linear plateau (σ_p_)*** is a parameter which was defined, according to [55], as the integral of the resulting stress between the initial maximum peak and the densification zone. This quantity indicates the energy absorption capacity (for instance, the longer and more stable the linear zone is, the better energy absorption capacity our model sample has).

(4) $$\sigma_{p}=\frac{\int_{\varepsilon_{0}}^{\varepsilon_{d}}\sigma (\varepsilon )d\varepsilon }{\varepsilon_{d}-\varepsilon_{0}}$$

***Average force for compression (F_m_)*** was defined as the ratio between the energy absorbed by the structure and the densification point value over which the compression is achieved [56]. Here, compression is relevant only up to the point of densification, denoted by *d_s_*, if it is calculated according to the force.

(5) $$F_{m}=\frac{1}{d_{s}}\int_{0}^{d_{s}}F\left(x\right)dx$$

If we consider the average stress that occurs after the compression, the equation takes the following form:

(6) $$\sigma_{m}=\frac{1}{\varepsilon_{d}}\int_{0}^{\varepsilon_{d}}\sigma (\varepsilon )d\varepsilon$$

***Compression load efficiency (CLE)*** was defined as the ratio between the average stress and the maximum stress value, specific to the maximum peak in the first portion of the compression curve. The value of the average stress was calculated by dividing the absorbed energy with the value of the load on which it was absorbed, this being equal to the value of the densification point [21].

(7) $$CLE=\frac{\sigma_{m}}{\sigma_{v}}$$

***Energy absorption efficiency (η_abs_)*** is a parameter defined as the ratio between the energy absorbed by a honeycomb structure up to the point of densification to the maximum theoretical energy that can be eventually absorbed [57]. The maximum energy represents the area under the curve formed by the constant stress value, from L/L_0_ = 0 to L/L_0_ = 1. This area has the shape of a rectangle with length 1 and width σ_v_ (the maximum stress value for the peak before the linear zone). For a clearer understanding, the above-mentioned parameters are drawn in Figure S5 in the Supporting Information.

(8) $$\eta_{abs}=\frac{E_{abs}}{\sigma_{v}\cdot 1}$$

## 3. Results and Discussion

**Figure 1:**
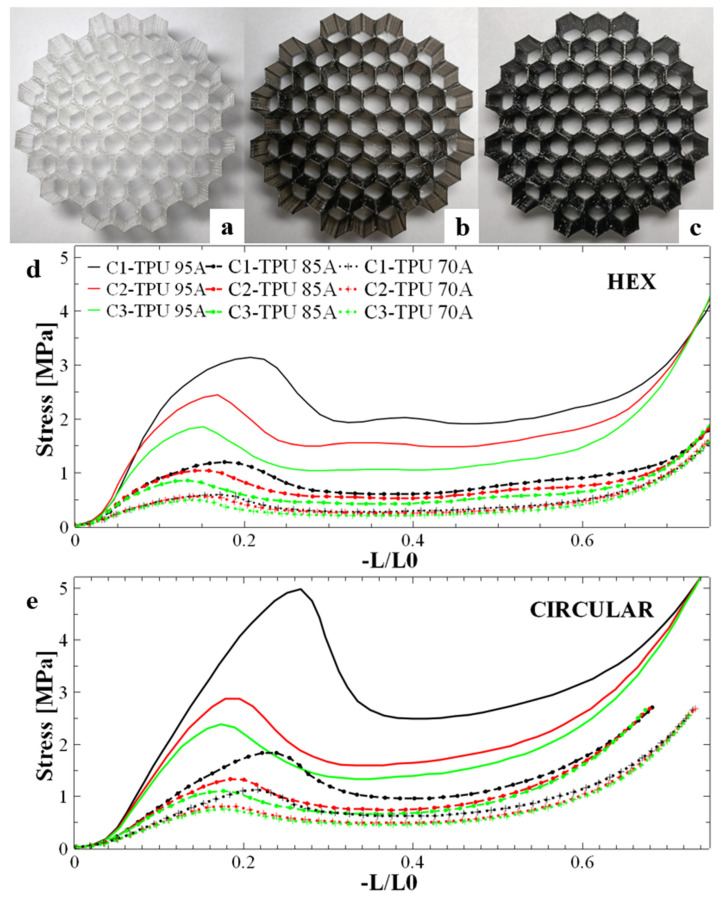
(**a**–**c**) Honeycomb-type energy-absorbing structures with hexagonal holes based internal configuration. The structures were 3D-printed using TPU 70A (**a**), TPU 85A (**b**) and TPU 95A (**c**) at a printing temperature of 215 °C. (**d**,**e**) First compression curve (C1), second compression curve (C2) and third compression curve (C3) of honeycomb absorbent structures with internal hexagonal (**d**) and circular (**e**) holes made using TPU 95A, TPU 85A and TPU 70A materials, respectively.

We begin our discussion by presenting, in Figure 1a–c, the physical appearance of MEAS honeycomb-like hexagonal structures 3D-printed from TPU 70A, TPU 85A, and TPU 95A materials, respectively, using a printing temperature of 215 °C. These structures, each with atop surface area of 440.5 mm^2^, were subjected to mechanical testing to evaluate their energy absorption capabilities and determine their degree of reusability. Specifically, all MEAS samples underwent compression tests by placing each specimen between two perfectly flat metal plates-one fixed and the other pushed by the device’s arm—ensuring that the pressing force was uniformly distributed over the contact surface. Three compression tests were conducted for each energy-absorbing honeycomb structure (photographs of representative samples at key positions are shown in Figures S6–S13 in the Supporting Information). Compression results for all three 3D-printed honeycomb hexagonal structures (HEX) are presented in Figure 1d, where stress is plotted as a function of strain rather than force versus compression distance. The results demonstrate a clear dependence of absorption capacity on the material type. Specifically, the maximum stress supported by the structure during the first compression correlates directly with the material’s hardness. TPU 95A exhibited the highest stress resistance, followed by TPU 85A, and then TPU 70A, which showed progressively lower stress resistance. This higher stress value also corresponds to a greater amount of energy absorbed by the structure. Furthermore, the samples exhibited a consistent decrease in stress between specific curves from one compression cycle to the next, indicating that the stress for a given sample is inversely proportional to the number of compressions. The difference between compression cycles also decreased progressively based on the material hardness, with TPU 95A exhibiting the largest differences between compressions and TPU 70A the smallest.

TPU hardness significantly influences the balance between peak stress, plateau stability, and elastic recovery. The harder TPU (95A) achieves higher peak stress and a more stable plateau while maintaining useful recoverability. The intermediate hardness (85A) represents a compromise between strength and ductility, whereas the softest grade (70A) exhibits the lowest peak stress but the greatest apparent elastic rebound. These trends reflect the competing effects of bulk polymer stiffness and interlayer adhesion in filament-assembled structures. To determine whether other honeycomb-like configurations could provide a better stress response than the hexagonal honeycomb structures, we 3D-printed honeycomb structures with a circular configuration (CIRCULAR), as shown in Figure S8 in the Supporting Information. These circular samples, with a top plane surface area of 737 mm^2^, exhibited behavior under compression similar to that of the hexagonal samples: the initial stress in the compression curves decreased with an increasing number of compressions (Figure 1e). Nonetheless, the shape of each curve corresponding to the first compression for all three honeycomb circular structures and all three types of materials differed from those corresponding to the second and third compressions. For instance, the absorption curves exhibited a steeper initial slope compared to the structures with hexagonal holes, leading to a well-defined peak with higher values. Additionally, the difference between the maximum stress value and the stress value in the plateau region was more pronounced during the first compression for all these samples. For this reason, we considered that part of the internal circular structure might have been damaged after the first compression, causing the subsequent two compressions to withstand lower stress. This hypothesis was further confirmed when visualizing optical photographs of the compressed honeycomb circular structures (see Figure S9 in the Supporting Information). The structure showed partial or complete ruptures of the walls in their thinnest areas. As shown in Figure S1a, the wall thickness was not uniform in the circular configuration. In contrast, the circular-cell configuration contains thin regions that act as stress concentrators, initiating localized cracks and leading to a more abrupt, fracture-like failure mode. Thus, the hexagonal geometry with uniform wall thickness converts incoming energy into distributed folding and buckling of cell walls, whereas the circular cells concentrate strain and fail locally.

In conclusion, we observed that the base structure with hexagonal holes (and especially structures produced from TPU 95A) exhibited the best overall performance in terms of both energy absorption efficiency and reusability across multiple compressions. Moreover, as shown in Figure S7 in the Supporting Information, the hexagonal MEAS sample maintained its original integrity (upon visual inspection), appearing undamaged after compression testing. Additionally, the curves presented in Figure 1d demonstrated the best plateau linearity, a crucial property for energy absorption. Furthermore, the differences between the first and second compressions were significantly smaller compared to the curves of the circular structures presented in Figure 1e. These characteristics pointed towards the hexagonal sample as being the most promising structure for further study.

**Figure 2:**
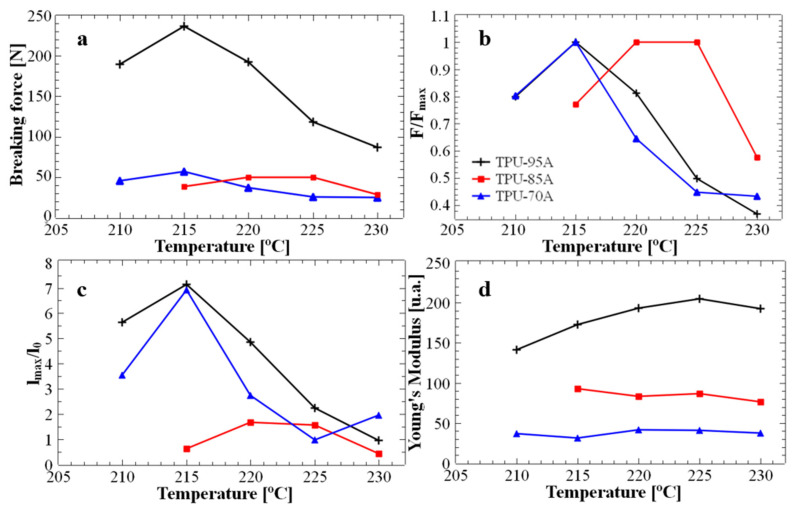
The main mechanical properties of TPU 95A, TPU 85A and TPU 70A standard ASTM-D628-10 Type V samples 3D-printed at various printing temperatures, as inferred from standard tensile strength measurements: ultimate breaking force (**a**), normalized force (**b**), maximum elongation (**c**) and Young’s modulus (**d**).

It is important to note that the printing temperature used to produce the above-mentioned 3D honeycomb structures was not randomly chosen, but it was previously optimized by fabricating and testing standard samples of TPU 95A, TPU 85A and TPU 70A. Figure 2 displays the main mechanical properties of all 3D-printed standard ASTM-D628-10 Type V samples, as inferred from typical mechanical tensile strength measurements. We aimed to study the properties of these standard samples in order to analyze how the printed layers adhere to each other at different printing temperatures. Our objective was to determine the optimal temperature for producing energy absorption honeycomb structures while ensuring their maximum structural resistance. First of all, Figure 2a shows that the TPU 95A standard structure exhibited a much higher maximum breaking force (at least double at higher printing temperatures, and more than three-fold higher at lower printing temperatures) when compared to the other two TPU 85A and TPU 70A structures. Secondly, when further normalizing the maximum forces specific to each temperature, as shown in Figure 2b, it was observed that the maximum breaking force for the TPU 70A and TPU 95A structures occurred at 215 °C. In contrast, for the TPU 85A structure, the maximum breaking force was observed at printing temperatures ranging between 220 °C and 225 °C. These results indicated that, in most cases, samples printed at 215 °C exhibited the best interlayer adhesion and higher overall print quality, as reflected by the superior force at which the samples failed. Furthermore, to study the final quality of the polymeric samples after exposure to different printing temperatures (considering that excessive temperatures could lead to material degradation), we determined the maximum elongation for all standard tensile specimens. This characteristic is important for reusable materials because it may indicate their ability to return to their original shape. Figure 2c depicts the dependence of elongation on printing temperature and clearly highlights that TPU 70A and TPU 95A structures printed at 215 °C displayed a maximum elongation of approximately 700%, which is more than seven times higher than the maximum elongation of 75% observed for the TPU 85A structure at the same temperature. For TPU 85A material, the maximum elongation (150%) was achieved at printing temperatures around 220–225 °C. Finally, Figure 2d emphasizes the variation in Young’s modulus corresponding to each ASTM-D628-10 Type V sample with printing temperature. While the Young’s modulus remained constant for TPU 70A and TPU 85A across all printing temperatures, it varied with temperature for TPU 95A. For example, an increase of about 15% in Young’s modulus was observed when increasing the printing temperature from 210 °C to 225 °C. A lower value of Young’s modulus may indicate increased elasticity. Note that we have chosen to determine Young’s modulus in arbitrary units because all utilized standard TPU samples were of the same size and for the purpose of solely finding the most appropriate printing temperature that could generate highly elastic standard samples, an exact value of the modulus was not necessary.

**Figure 3:**
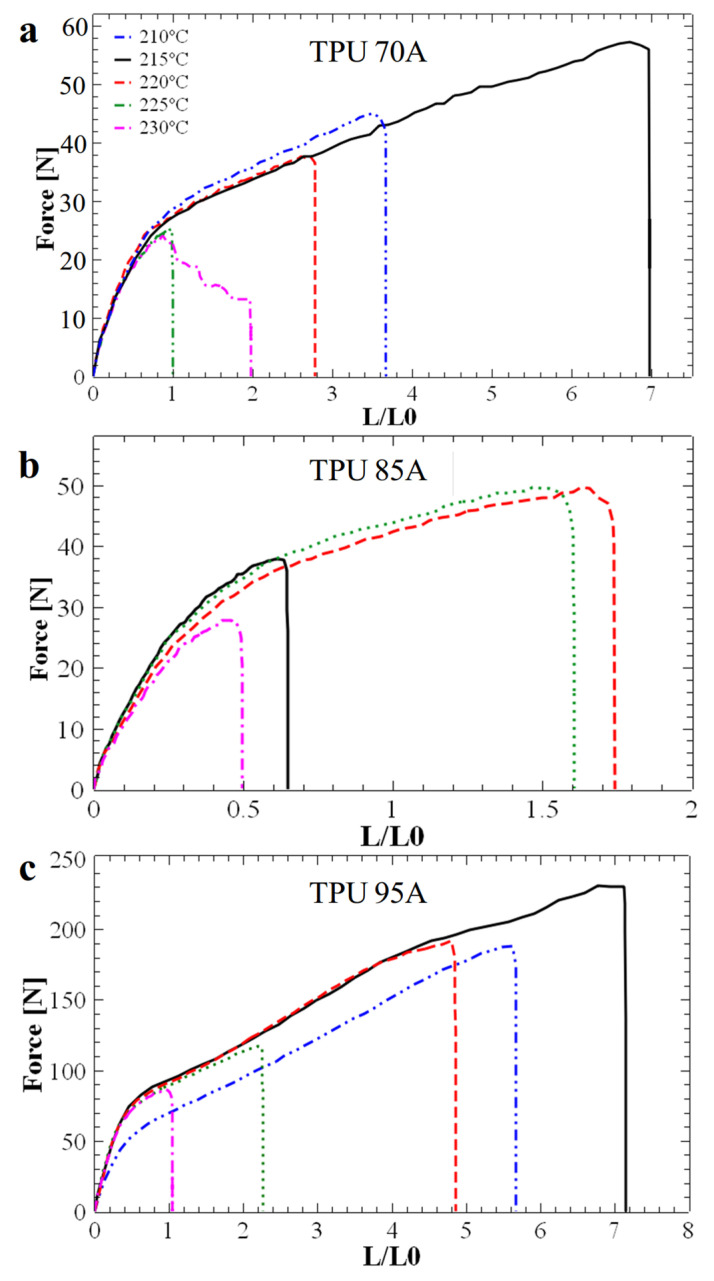
Interlayer force-elongation curves for standard ASTM-D628-10 Type V samples of TPU 70A (**a**), TPU 85A (**b**) and TPU 95A (**c**) obtained by 3D printing at temperatures varying from 210 °C to 230 °C in increments of 5 °C.

The data presented in Figure 2 were extracted from the force-elongation curves shown in Figure 3. These curves display the critical force and elongation values up to the point of sample failure and help determine the optimum printing temperature. While the initial goal of this study was to optimize printing parameters for flexible materials such as TPU 95A, the subsequent objective was to observe the maximum force and elongation of samples printed at different temperatures to establish the optimal printing temperature for achieving the most efficient layer adhesion. Figure 3 shows that the typical shape of the interlayer force-elongation curve indicates an optimal printing temperature of 215 °C for the TPU 70A and TPU 95A materials. These materials withstand a maximum breaking interlayer force of approximately 55 N (Figure 3a) and 225 N (Figure 3c), respectively. Instead, the TPU 85A standard sample displayed a maximum breaking interlayer force slightly below 50 N at a higher printing temperature of 220 °C (Figure 3b). Under these conditions, and knowing that the hardness of TPU 85A lies between that of TPU 70A and TPU 95A, and to optimize the printing process, we chose to 3D-print all our honeycomb (hexagonal and circular) structures at 215 °C. For example, although the Young’s modulus of TPU 95A at 215 °C is lower, its corresponding elongation and breaking force are higher, which is more relevant for mechanical energy-absorbing applications.

**Figure 4:**
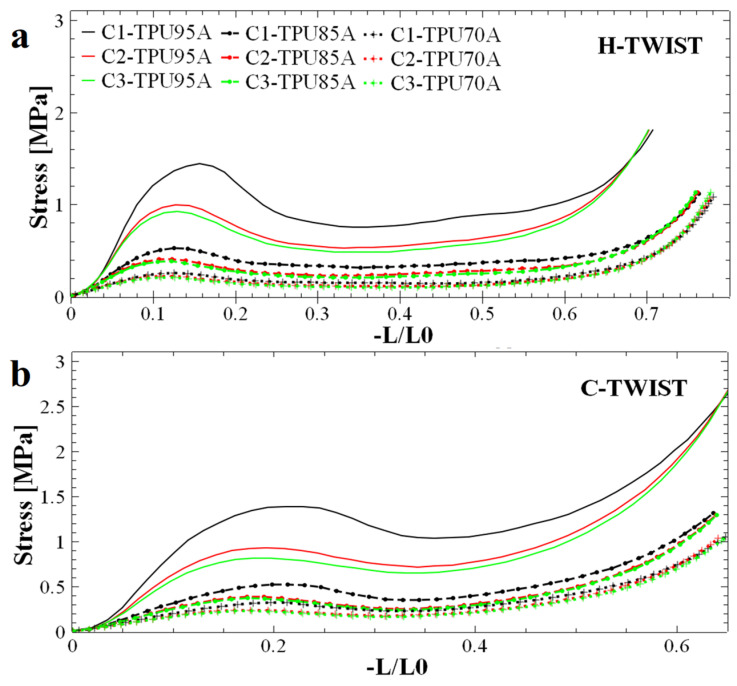
Compression curves corresponding to honeycomb absorbent structures with internal hexagonal (**a**) and circular (**b**) holes that display the top plane twisted at an angle of 30°. Structures were made using TPU 95A, TPU 85A and TPU 70A materials, respectively.

To investigate how twisting the honeycomb absorbent structures with internal hexagonal and circular holes would impact their performance in terms of energy absorption upon compression, we rotated both hexagonal and circular structures by 30°. Figure 4 illustrates the behavior of the twisted structures with internal hexagonal holes (Figure 4a) and circular holes (Figure 4b) during compression, using the same materials for production. A general characteristic of twisted structures is an offset between compressions, with the curve for the first compression showing a noticeably higher stress value than those for the two subsequent compressions. Since these latter compressions display similar values, we anticipate that further compressions will also exhibit comparable stress values. Therefore, we assume that the samples discussed here could withstand a series of additional compressions before failure. Moreover, although this characteristic of rotated structures may enhance their reusability potential, we observed that in the case of both basic (Figure 1e) and twisted (Figure 4b) structures with circular holes, the compression curve shows a reduced linear area compared to the hexagonal hole structures (Figure 1d and Figure 4a). This suggests lower efficiency in absorbing mechanical energy (the efficiency parameter, as you can see in Equation (8), is highly dependent on the length of the linear plateau; a longer linear area allows a larger amount of energy to be absorbed).

In the case of the twisted hexagonal structure (Figure 4a) the height difference between the maximum peak and the partially linear area was smaller than that of the non-twisted hexagonal hole structure (Figure 1d). This could indicate that twisting the structures improves their crushing load efficiency (CLE), as presented in Equation (7). This parameter depends on two main stress values: the maximum peak and the average stress. For example, if the maximum peak and plateau stress values are more similar, the CLE parameter will be closer to 1. Additionally, the shock absorption mechanism remains consistent because the gap between the peak and plateau is reduced. Therefore, we can conclude that the compression behavior of hexagonal cell structures is more balanced compared to structures with circular holes. In the case of twisted hexagonal cells, some crushing parameters may be superior. Nonetheless, from a quantitative point of view, the non-twisted structures (Figure 1d,e) can withstand significantly higher peak stress compared to the twisted structures (Figure 4a,b). This difference could translate into a greater amount of absorbed mechanical energy.

Besides its dependence on the length of the linear plateau, the efficiency parameter also depends on the difference in height between the maximum peak and the linear area. When this difference is small, the absorbed energy approaches the total energy that can theoretically be absorbed. For example, comparing Figure 1 and Figure 4 reveal that the basic structures with circular and hexagonal holes absorb a larger amount of energy compared to the twisted structures. Moreover, this amount of potentially absorbed energy seems also to be dependent on the type of TPU material used.

**Figure 5:**
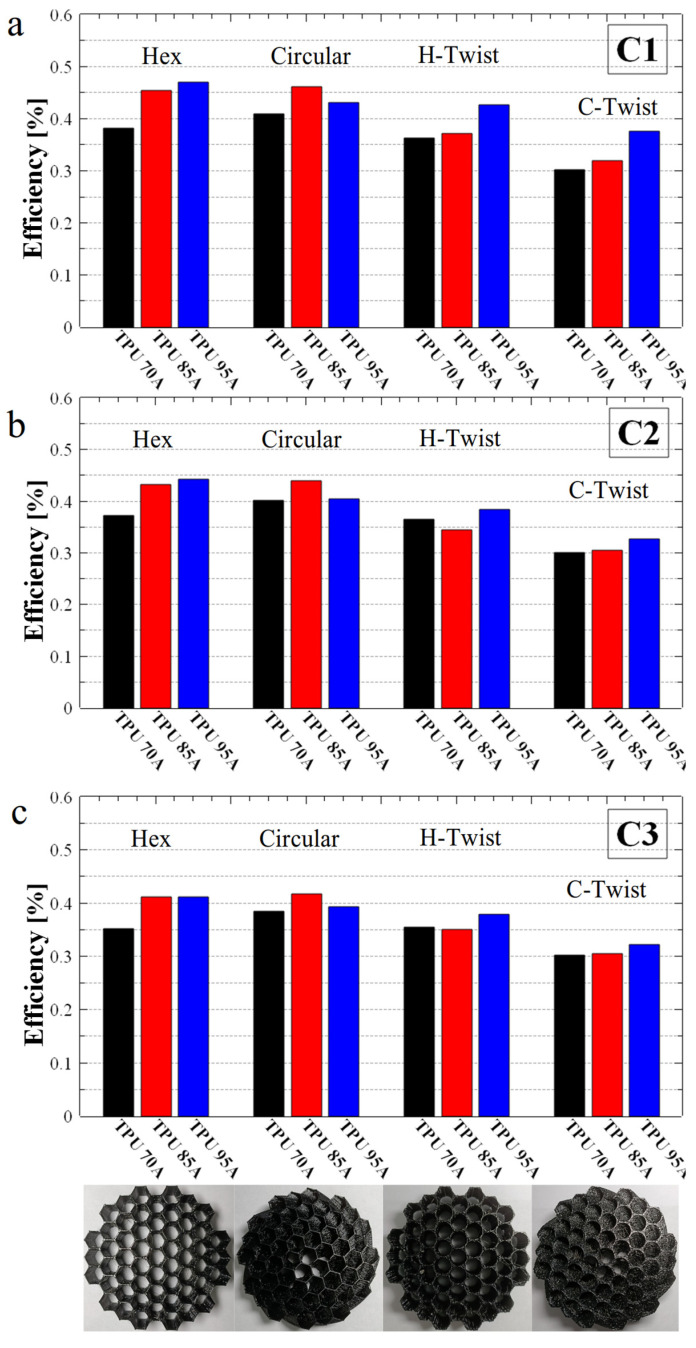
Summary emphasizing the energy absorption efficiency (η_abs_) of all structures subjected to one (**a**) two (**b**) and three (**c**) compressions, respectively. At the bottom of the figure, we also present photographs of hexagonal, hexagonal twist, circular and circular twist samples produced from TPU 95A.

To generate a fair comparison of all samples in terms of the efficiency in absorbing mechanical energy during compression, the efficiency for each compression cycle was compiled separately, and the results are presented in Figure 5. A thorough analysis of these results indicates that most samples exhibited relatively similar energy absorption efficiencies, ranging between 36% and 47%. The highest absorption efficiency was achieved by the basic hexagonal hole structure made from TPU 95A (see the blue bar in Figure 5a), followed by the 30° twisted and non-twisted honeycomb with hexagonal internal patterns made from TPU 85A (see the red bar in Figure 5a). However, when considering how well the structure maintains its absorption capacity over repeated compressions, the best-performing structure was the hexagonal hole structure rotated by 30° and made of TPU 85A (see the red bar in Figure 5a), which showed a smaller variation in specific efficiencies across compression cycles. Nonetheless, the overall difference in the efficiency of energy absorption was rather small when comparing these three samples.

We further observed from the specific efficiency histograms presented in Figure 5 that, overall, the twisted hexagonal structures exhibited similar or slightly better efficiency compared to non-twisted ones in some cases. Additionally, energy absorption efficiency depended on material hardness. For softer materials like TPU 70A, energy absorption was more efficient in twisted hexagonal structure. In contrast, for harder materials such as TPU 85A and TPU 95A, the hexagonal non-twisted structures demonstrated higher energy absorption efficiency. Moreover, Figure 5 clearly shows that honeycomb structures with a hexagonal internal configuration (twisted or not) outperform those with a circular internal structure (twisted or not) in mechanical energy absorption. So, in terms of energy absorption efficiency, the non-twisted hexagonal hole structure demonstrated the highest capability as an energy absorber. And, because this structure made from TPU 95A exhibited an absorption efficiency exceeding 47%, we consider this sample the best outcome of our present study.

**Figure 6:**
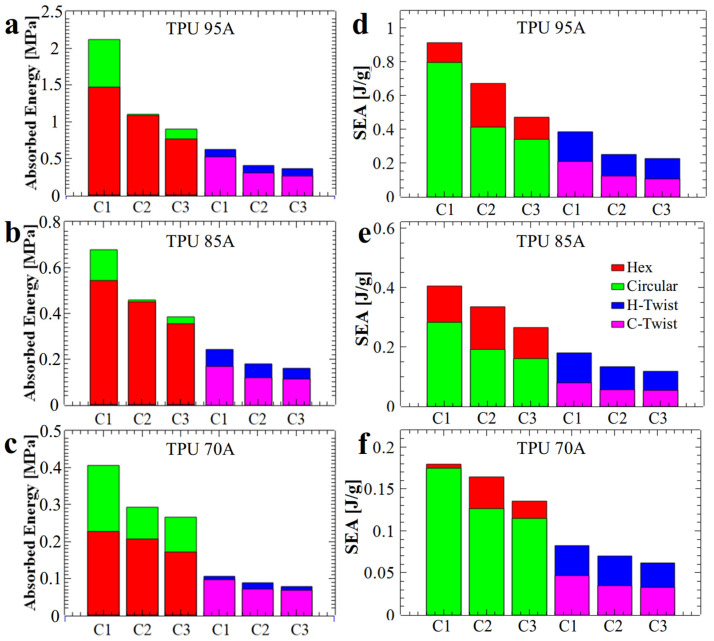
The quantity of energy absorbed (**a**–**c**) and specific energy absorption (**d**–**f**) by honeycomb structures produced using TPU 95A (**a**,**d**), TPU 85A (**b**,**e**) and TPU 70A (**c**,**f**) materials, respectively.

Furthermore, because each MEAS sample may vary in density, water absorption, and printing parameters, the samples were weighed. We used mass to calculate the specific energy absorption (SEA), as this parameter quantifies the energy stored per unit mass. In terms of energy absorption, Figure 6a–c demonstrates that structures with circular holes have absorbed a greater amount of energy as compared to their hexagonal and twisted analogs. However, the SEA coefficient changed because samples with circular holes required more material to produce (Figure 6d–f). Therefore, we can confidently state that structures with hexagonal holes absorbed a smaller amount of energy, but the latter was absorbed more efficiently, as in this case the mass of utilized material was smaller. For example, structures with circular holes used between 16 g and 20.5 g of material, while those with hexagonal holes required only 10 g to 14 g. To illustrate, with approximately the same amount of material, two circular-hole structures or three hexagonal-hole structures could be produced; notably, the total energy absorbed by three hexagonal-hole honeycomb structures exceeded that absorbed by two structures with circular holes. Moreover, the printing process for hexagonal hole structures was faster and of higher quality because the wall thickness was achieved in a single pass of the extruder, compared to circular hole structures where the extruder made a series of extra moves. In terms of price, a quick cost analysis has revealed that 20 g of TPU 95A sample cost under 1 euro, while approximately 1.5 euros must be paid for the same amount of TPU 85A, and around 1.6 euros for TPU 70A.

Additionally, the time to print samples with circular holes was twice that necessary to print structures with hexagonal holes, although this typically depends also on the type of utilized polymeric material. Furthermore, we observed that in the case of the structure with circular holes and made from the softer TPU 70A material, the amount of absorbed energy for the first compression was much lower (approximately 0.4 MPa, see Figure 6c), compared to that of the hardest TPU 95A material (approximately 2.1 MPa, see Figure 6a). We also noticed in Figure 6a–c that the basic structures with circular holes (green bars) experienced a significant difference in energy absorption between the first and second compressions, compared to the structures with hexagonal holes (red bars). Specifically, the first compression of the circular hole structures showed much higher energy absorption than that of the hexagonal-hole structures, whereas the second compression resulted in similar energy absorption levels for both types of structures. This aspect was more pronounced in structures printed from harder materials: a difference of approximately 0.1 MPa between C1 and C2 was measured for TPU 70A (Figure 6c), while a difference of approximately 1 MPa was determined in the case of TPU 95A (Figure 6a).

After being compressed three times, the physical appearance of all structures remained similar to the original one. This can be visualized in Figure S7 in the Supporting Information. Moreover, for structures with hexagonal holes, the walls visually retained their shape and linearity after compressions. In particular, structures with circular made from TPU 95A and subjected to three compressions experienced total or partial breaks in the walls at the thinner, intentionally designed areas. However, structures printed with a 30° twist angle showed no physical alterations after compressions.

**Figure 7:**
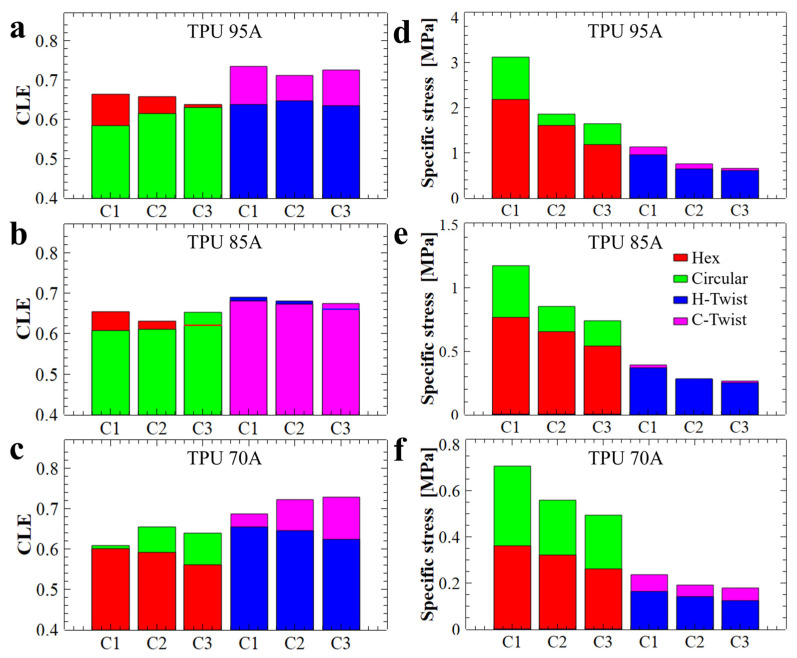
The crushing load efficiency parameter (**a**–**c**) and the specific stress of the linear plateau (**d**–**f**) specific to each honeycomb structure produced from TPU 95A (**a**), TPU 85A (**b**) and TPU 70A (**c**) materials, respectively.

To quantitatively evaluate the mechanical energy absorption efficiency of the investigated structures, the dimensionless CLE parameter was computed and analyzed. A CLE value approaching unity is indicative of superior energy absorption characteristics. As shown in Figure 7a–c, the twisted structures (H-twist and C-twist) exhibited, on average, higher CLE values, i.e., values closer to 1. In comparison the CLE values corresponding to the non-twisted counterparts were generally lower (except possibly for the hexagonal structures made from TPU 95A), i.e., further away from 1. Introducing a twist reduces the instantaneous peak stress and total absorbed energy but increases crushing load efficiency (CLE). This behavior is consistent with a transition from abrupt cell wall engagement to a more gradual, distributed deformation. Therefore, twisting promotes progressive loading of successive cell walls, improving CLE at the expense of initial load-bearing capacity.

In the case of structures with hexagonal holes, a decrease in the CLE parameter was observed with repeated compressions. This behavior is attributed to the fact that, although the compression curves maintained their general shape across successive cycles (Figure 1d), the maximum force values progressively decreased. This trend can be explained by both the structural configuration and the fabrication method employed. Specifically, in structures with circular holes, the initial slope of the compression curve (Figure 1e) was lower compared to that of the hexagonal-hole structures (Figure 1d), indicating a different stress absorption mechanism. A steeper slope typically reflects a more rigid structure that maintains stiffness up to the peak load before yielding, whereas a gentler slope suggests a structure that absorbs stress more gradually before failure.

From a manufacturing perspective, structures with circular holes are more susceptible to defects due to the increased complexity involved in 3D printing flexible filaments into such geometries. These larger defects, combined with inherent variations in wall thickness, contribute to structural rearrangement during the initial compression cycle, as indicated by the differences observed between the first (C1) and second (C2) compression curves (Figure 1e). The analysis of the CLE histograms presented in Figure 7a–c revealed that the highest CLE values, ranging from 72% to 73.5%, were recorded for twisted structures with circular hole fabricated from TPU 95A (Figure 7a) and TPU 70A (Figure 7c), respectively. In contrast, the hexagonal holes structures produced from TPU 95A exhibited a CLE coefficient of 0.665 (i.e., 66.5%, Figure 7a), while the H-twist structure fabricated from TPU 85A displayed a CLE value of 0.69 (i.e., 69%, Figure 7b). Although the Hex structure demonstrated outstanding performance across the previously evaluated parameters, its CLE value did not reach a higher level. Nevertheless, the recorded value remained very close to the theoretical maximum, confirming the structure’s overall superior energy absorption behavior.

To characterize the linearity of a specific absorption plateau, we further calculated the specific stress of that plateau. The results are presented in Figure 7d–f. It is important to emphasize that the energy absorption capability of a structure is strongly influenced by both the linearity and extent of the plateau region observed during compression. A longer and more stable plateau allows the structure to absorb greater amounts of mechanical energy efficiently, as the stress is distributed uniformly over an extended range of deformation. In particular, structures that maintain near-constant stress across the plateau phase exhibit improved mechanical performance, minimizing localized failures and optimizing energy dissipation. Therefore, designing honeycomb structures with geometries and material properties that promote a long, stable plateau is essential for developing efficient, reusable energy-absorbing systems. For example, the stress values of the basic samples (Hex and Circular) decreased considerably after each compression (see Figure 7d), whereas in the case of structures twisted by 30°, the stress values decreased much less after each compression. Thus, we conclude that twisted structures with hexagonal holes are capable of preserving the linear plateau stress value at a level comparable to that observed during the initial compression.

Nonetheless, when considering parameters such as the energy absorption efficiency shown in Figure 5, as well as the CLE parameter depicted in Figure 7a, the structure with hexagonal holes made from TPU 95A demonstrated the best overall energy absorption properties. The energy absorbed by this structure was 1.5 MPa in terms of stress and 0.91 J/g in terms of specific absorbed energy. Thus, TPU 95A appears to be a more versatile material, capable of exhibiting a wide range of the maximum compression force values. Alongside the TPU 95A, another structure that efficiently absorbs mechanical energy is the twisted structure with hexagonal holes, produced from TPU 85A. This structure demonstrated an energy absorption efficiency of 46% (Figure 5a), while its corresponding CLE parameter, as compiled from Figure 7b, was 0.69 (i.e., 69%). However, this structure absorbed only 0.2 MPa of energy (as shown in Figure 6b) and therefore appears to be suitable only for absorbing low-amplitude mechanical shocks. Similar properties were also observed for this type of structure when produced from TPU 95A.

In the case of twisted structures with circular holes, although they exhibited a favorable CLE parameter (see Figure 7a–c), they absorbed only a small amount of specific energy relative to their weight (see Figure 6d–f). Furthermore, the efficiency results for these structures (see Figure 5) were lower than those of the basic structures. Therefore, we decide not to pursue twisted structures with circular holes further in our study.

In conclusion, the combined results obtained in this study clearly indicate that structures with hexagonal holes are the most effective configurations for developing energy-absorbing products. The findings also suggest that TPU 95A is the most versatile material for producing structures with hexagonal holes. Therefore, for subsequent studies, we produced a set of four basic, non-twisted structures with hexagonal holes using TPU 95A (with four samples per set to ensure experimental reproducibility).

**Figure 8:**
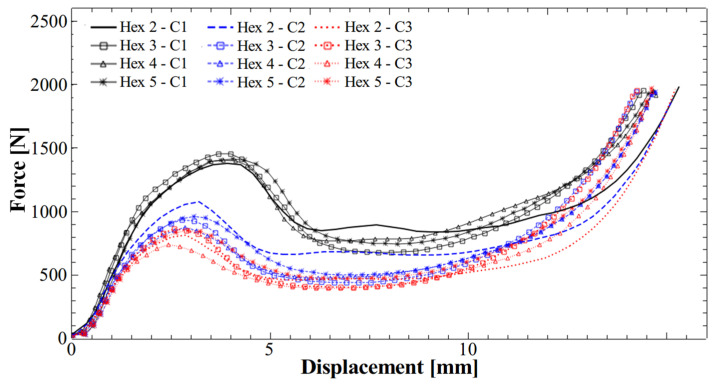
Compression curves of the newly produced honeycomb energy-absorbent structures with hexagonal holes (Hex 2–5) and made of TPU 95A.

Figure 8 shows that the specific compression curves for structures with hexagonal holes remained consistent across all four samples, indicating good reproducibility. Additionally, all compression curve profiles were smooth, suggesting that the structures did not exhibit successive wall failures, even after three compression cycles. In contrast, compression curves reported in similar studies exhibited multiple peaks within the linear plateau region [13], suggesting the compression was accompanied by gradual wall destruction.

**Figure 9:**
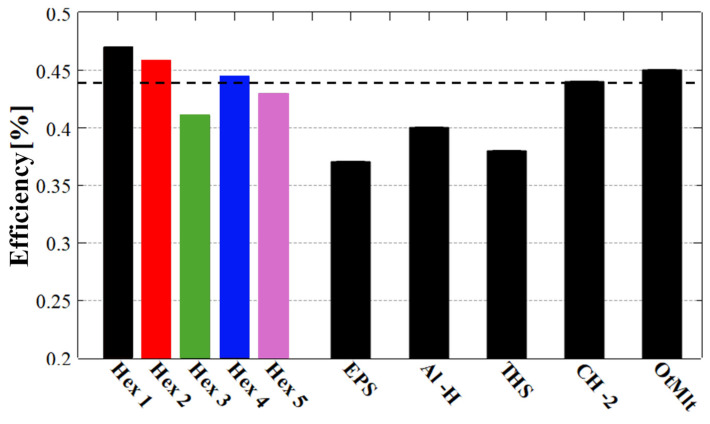
Energy absorption efficiency (η_abs_) extracted from structures with hexagonal holes made of TPU 95A after the first compression. Hex2–Hex5 represent samples made later, to verify the results obtained for the initial sample Hex1. The average of the five Hex samples is represented in the figure as a dashed line. In comparison, in the following columns on the right we represent the values of η_abs_ provided in the literature for expanded polystyrene foam (EPS) [46], aluminum honeycomb (Al-H) [58], twin hemispheres Skydex (THS) [59], CH-2 [21], and octet truss macrolattice (OtMlt) [57]).

To further evaluate the practical viability of the basic hexagonal structure, we extracted and analyzed the energy absorption efficiencies (η_abs_) from the compression curves corresponding to each of the four newly fabricated samples after their first compression cycle. For a comprehensive comparison, we also included the η_abs_ value calculated from the initial hexagonal structure made from TPU 95A (denoted as Hex 1, based on the curves shown in Figure 1d). These results were then compared with values reported in the literature for other energy-absorbing systems, as illustrated in Figure 9.

Obviously, one of our goals was to obtain η_abs_ values equal to or greater than those already reported for various energy absorption structures, including those manufactured using similar 3D printing methods or advanced, yet costly, techniques. Note that in the data used for comparison we also included structures that were reported as destroyable after the first compression.

Figure 9 reveals that the structures fabricated in this work are superior in terms of η_abs_ compared to available commercial absorption structures. For example, although the average η_abs_ values of our samples were similar to those reported for the CH-2 structure [10], our structures neither failed after the first compression nor were produced using an expensive fabrication method, as was the case for the CH-2 structure.

Moreover, our results for 2D structures with hexagonal holes generally align with those obtained for octet truss macrolattice structures [39]. The latter are 3D structures (such as TPMSor beam structures) that involve variations in shape along the sample’s growth direction and are more challenging to produce using FDM 3D printing methods. This difficulty arises because the printer’s precision depends on the nozzle size, with a typical standard nozzle diameter of 0.4 mm, which limits the resolution of finer details. Furthermore, considering the production method, the results obtained here are promising, as the approach used in this work is more cost-effective and offers considerable scalability potential compared to techniques employed in studies involving significantly more complex structures, which currently may have limited applicability for mass production.

Finally, when comparing our structures to other similar honeycomb structures reported in the literature [28], we observed that average and maximum η_abs_ values of 0.44 and 0.47, respectively, are significantly higher than the reported value of 0.36. These differences can be attributed to the geometric dimensions of the basic cell and the testing setup. Specifically, we compressed our samples out-of-plane rather than in-plane, which allows for greater applicability by simply increasing the height and multiplying the basic cell or the thickness of the structure.

**Figure 10:**
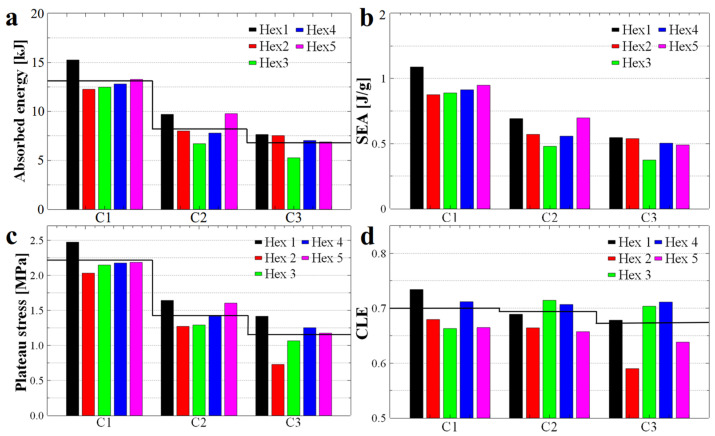
Absorbed energy (**a**), specific energy absorbed per unit mass (**b**), specific stress of the linear plateau (**c**) and CLE parameter (**d**) compiled for honeycomb structures produced using TPU 95A. Hex2–Hex5 represent the samples made later to verify the results and Hex1 is the initial sample. The black line indicates the average absorbed energy for each compression.

Figure 10a illustrates the histograms representing the energy absorbed by each sample after the first (C1), second (C2), and third (C3) compressions. The black line indicates the average absorbed energy. Following this line, it is immediately apparent that the greatest difference in absorbed energy (~4824 J) occurred between C1 and C2. The gap in absorbed energy between C2 and C3 was reduced to approximately ~1523 J, suggesting long-term cyclic stability and indicating the potential reusability of the structure.

Furthermore, when comparing SEA coefficient values (Figure 10b), they are similar to those reported in the literature [38], but superior to those of raw materials. Additionally, price of one gram of TPU 95A material is lower compared to the lighter materials used in other studies, making our structures more cost-effective and reusable. While the obtained SEA coefficient can be attributed to the material’s weight, we specifically selected this type of material for applications where the structures must withstand multiple mechanical shocks and avoid significant damage—that is, a flexible material capable of absorbing an increased amount of energy over time.

In the case of the linear plateau stress presented in Figure 10c, we observed a steady decrease caused by the three compressions. As shown in Figure 8, there are differences between the linear regions of the curves, which are also reflected in the values of the linear plateau stress. However, the overall shape of the curves remained similar across the different compressions, resulting in a trend of values comparable to that of energy absorption (compare Figure 10a with Figure 10c). In contrast, the average value of the CLE parameter (see the black line in Figure 10d) exhibits only minor variation between compressions, with some structures even displaying a higher CLE coefficient upon the second or third compression.

**Figure 11:**
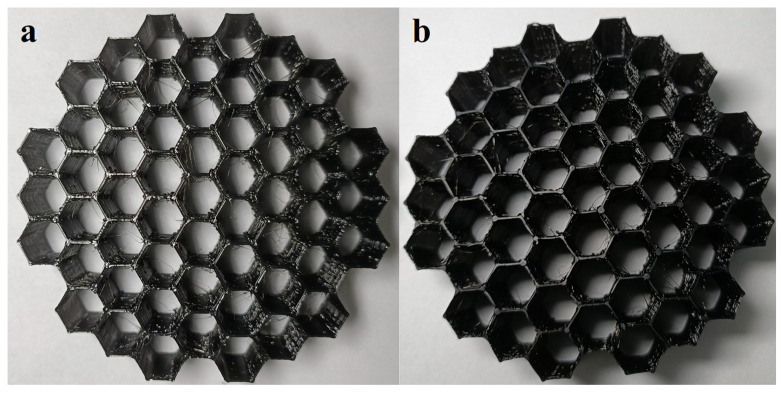
Photographs depicting a structure with hexagonal holes made of TPU 95A before (**a**) and after being subjected to three repeated compressions (**b**).

The CLE coefficient represents the difference between the linear stress plateau and the maximum peak preceding the plateau. Samples composed of elastic materials typically exhibit a noticeable difference between the peak and the linear region. Additionally, an energy-absorbing structure that is damaged during compression generally dissipates energy by breaking its layers, which can reduce the difference between the peak and the linear region. In our case, such damage is minimal and imperceptible to the naked eye, as demonstrated in Figure 11, where one of our structures is shown before and after repeated compressions. The only observable change is a slight expansion in the linearity of the structure’s walls. Furthermore, after a thorough visual inspection of structures subjected to repeated compressions, we concluded that all compressed structures were able to return to their initial shape, without significant structural differences.

Future investigations will extend this work to high-rate dynamic impact testing to evaluate performance under realistic collision conditions. Longer-term fatigue studies, involving more than three compression cycles, are necessary to establish the lifetime and serviceability for practical applications. Exploring alternative topologies (e.g., TPMS) and hybrid material designs (such as multi-material printing and fiber reinforcement) could further optimize the balance between energy absorption and reusability. Finally, ongoing finite element simulations combined with experimental data will clarify stress distributions and guide process optimization to enhance cyclic durability.

## 4. Conclusions

In this study, we systematically evaluated the mechanical energy absorption performance of various 3D-printed honeycomb structures fabricated from flexible thermoplastic polyurethanes (TPU 70A, TPU 85A, and TPU 95A) using out-of-plane compression tests to simulate real-world mechanical impacts. The investigation consisted of several stages that highlighted key findings and provided valuable insights into material selection, structural design, and manufacturing parameters.

We demonstrated that honeycomb structures with hexagonal holes exhibit superior mechanical performance compared to those with circular holes. The TPU 95A hexagonal structure achieved a peak efficiency (η_abs_) of 47% and maintained a CLE of 66.5%, demonstrating both high efficiency and stable load distribution. This structure also demonstrated excellent reusability and maintained structural integrity over repeated loading cycles, which is a significant advantage compared to traditional energy absorbers such as EPS foams. It is important to note that before conducting these experimental procedures, optimization testing of printing conditions was performed using ASTM-D628-10 Type V standard samples. The testing revealed that a printing temperature of 215 °C provided the best combination of maximum breaking force, elongation at break, and Young’s modulus across all tested TPUs. These parameters ensured optimal interlayer adhesion and enhanced the mechanical performance of the printed structures.

Furthermore, twisting the honeycomb structures by 30° resulted in a moderate increase in CLE, with the highest values observed in twisted structures featuring circular holes fabricated from TPU 95A (72%) and TPU 70A (73.5%). However, this improvement came at the cost of reduced peak stress and total absorbed energy, making the non-twisted designs with hexagonal hole designs more favorable for applications requiring high energy absorption capacity. Additionally, a SEA analysis confirmed that, while structures with circular holes absorbed greater absolute energy due to their larger mass, hexagonal holes structures were more efficient per unit of mass, making them more suitable for lightweight applications.

Finally, additional reproducibility tests conducted on four samples confirmed the robustness of the basic hexagonal hole design made from TPU 95A, achieving consistent energy absorption efficiency values that were close to or even exceeded those reported for more expensive or single-use structures fabricated using advanced additive manufacturing techniques.

Overall, this study demonstrates that optimized FDM printing provides a simple and cost-effective method for fabricating reusable TPU-based energy absorbers with performance levels comparable to or exceeding those of conventional single-use systems. Our structures represent a promising solution for applications in personal protection systems, automotive safety components, and sports equipment. They can also be applied in protective packaging for sensitive electronic components, impact-mitigation systems in transportation and logistics, and biomedical cushioning or orthotic devices where repeated load absorption and shape recovery are essential. In terms of price, the cost analysis revealed that TPU filaments are economically advantageous, with the average production cost of a 20 g TPU 95A sample being under 1 euro, approximately 1.5 euros for TPU 85A, and around 1.6 euros for TPU 70A. The simplicity and cost-effectiveness of the FDM manufacturing process make these structures attractive for scalable, sustainable deployment across multiple application domains. Future work will focus on further optimizing cell designs, investigating dynamic (high-speed) compression behavior, exploring alternative topologies (e.g., TPMS), expanding the range of materials tested beyond TPU variants to include hybrid material designs (such as multi-material prints and fiber reinforcement), and integrating these structures into practical engineering systems to enhance safety and performance.

## Figures and Tables

**Table 1:** Characteristics of the polymeric filaments as provided by the manufacturers elsewhere [46,47,48].

Material	Young Modulus [MPa]	Elongation [%]	Breaking Stress [MPa]	Density [g/cm^3^]	Printing Temperature [°C]
TPU 95A	67 ± 2	700	37.9 ± 1.6	1.22	220–235
TPU 85A	12	660	26	1.19	225–235
TPU 70A	32	900	45	1.08	215–235

## Data Availability

The data are contained within this article and Supplementary Materials; additional data are available on request from the corresponding author.

## Supplementary Materials

The following supporting information can be downloaded at: https://www.mdpi.com/article/10.3390/polym17223035/s1, Figure S1: The basic cell of the honeycomb, circle (a) and hexagon (b) structures; Figure S2: Honeycomb absorption structure with hexagonal holes (left) and circular holes (right); Figure S3: Honeycomb absorption structure with hexagonal holes (left) and circular holes (right), twisted with an angle of 30°; Figure S4: The printing method of standard ASTM-D628-10 Type V samples, so they can be used for layer adhesion testing; Figure S5: Absorption curve of a hexagonal holes TPU 95A structure, used to visualize the parameters *σ*_*v*_, *ε*_*d*_ and *ε*_0_; Figure S6: Honeycomb-type energy-absorbing structure with hexagonal holes internal configuration. The structures were 3D printed using: (a) TPU 70A, (b) TPU 85A and (c) TPU 95A; Figure S7: Honeycomb-type energy-absorbing structure with hexagonal holes internal configuration after undergoing a compression test involving three successive compressions. The structures were 3D printed using: (a) TPU 70A, (b) TPU 85A and (c) TPU 95A; Figure S8: Honeycomb-type energy-absorbing structure with circle holes internal configuration. The structures were 3D printed using: (a) TPU 70A, (b) TPU 85A and (c) TPU 95A; Figure S9: Honeycomb-type energy-absorbing structure with circle holes internal configuration after undergoing a compression test involving three successive compressions. The structures were 3D printed using: (a) TPU 70A, (b) TPU 85A and (c) TPU 95A; Figure S10: Honeycomb-type energy-absorbing structure with hexagonal holes, featuring the top plane twisted with an angle of 30°. The structures were 3D printed using: (a) TPU 70A, (b) TPU 85A and (c) TPU 95A; Figure S11: Honeycomb-type energy-absorbing structure with hexagonal holes, featuring the top plane twisted with an angle of 30°, after undergoing a compression test involving three successive compressions. The structures were 3D printed using: (a) TPU 70A, (b) TPU 85A and (c) TPU 95A; Figure S12: Honeycomb-type energy-absorbing structure with circular holes, featuring the top plane twisted with an angle of 30°. The structures were 3D printed using: (a) TPU 70A, (b) TPU 85A and (c) TPU 95A; Figure S13: Honeycomb-type energy-absorbing structure with circular holes, featuring the top plane twisted with an angle of 30°, after undergoing a compression test involving three successive compressions. The structures were 3D printed using: (a) TPU 70A, (b) TPU 85A and (c) TPU 95A.

## References

[1] Bates F.S., Fredrickson G.H. (1999). Block Copolymers—Designer Soft Materials. Phys. Today.

[2] Ditte K., Perez J., Chae S., Hambsch M., Al-Hussein M., Komber H., Formanek P., Mannsfeld S.C.B., Fery A., Kiriy A. (2021). Ultrasoft and High-Mobility Block Copolymers for Skin-Compatible Electronics. Adv. Mater..

[3] Handrea-Dragan M., Botiz I. (2021). Multifunctional Structured Platforms: From Patterning of Polymer-Based Films to Their Subsequent Filling with Various Nanomaterials. Polymers.

[4] Leordean C., Marta B., Gabudean A.-M., Focsan M., Botiz I., Astilean S. (2015). Fabrication of highly active and cost effective SERS plasmonic substrates by electrophoretic deposition of gold nanoparticles on a DVD template. Appl. Surf. Sci..

[5] Darko C., Botiz I., Reiter G., Breiby D.W., Andreasen J.W., Roth S.V., Smilgies D.-M., Metwalli E., Papadakis C.M. (2009). Crystallization in diblock copolymer thin films at different degrees of supercooling. Phys. Rev. E.

[6] Jahanshahi K., Botiz I., Reiter R., Thomann R., Heck B., Shokri R., Stille W., Reiter G. (2013). Crystallization of Poly(γ-benzyl <scp>l</scp> -glutamate) in Thin Film Solutions: Structure and Pattern Formation. Macromolecules.

[7] Botiz I., Codescu M.-A., Farcau C., Leordean C., Astilean S., Silva C., Stingelin N. (2017). Convective self-assembly of π-conjugated oligomers and polymers. J. Mater. Chem. C.

[8] Thomas T., Tiwari G. (2019). Crushing behavior of honeycomb structure: A review. Int. J. Crashworthiness.

[9] Asprone D., Auricchio F., Menna C., Morganti S., Prota A., Reali A. (2013). Statistical finite element analysis of the buckling behavior of honeycomb structures. Compos. Struct..

[10] Ainin F.N., Azaman M.D., Abdul Majid M.S., Ridzuan M.J.M. (2025). Mechanical behavior, energy absorption, and failure mechanism of 3D-printed hexagonal honeycomb core under dynamic and quasi-static loadings. Polym. Compos..

[11] Wu Y., Fang J., Wu C., Li C., Sun G., Li Q. (2023). Additively manufactured materials and structures: A state-of-the-art review on their mechanical characteristics and energy absorption. Int. J. Mech. Sci..

[12] Zhang Q., Yang X., Li P., Huang G., Feng S., Shen C., Han B., Zhang X., Jin F., Xu F. (2015). Bioinspired engineering of honeycomb structure—Using nature to inspire human innovation. Prog. Mater. Sci..

[13] Lu G., Yu T. (2003). Composite materials and structures. Energy Absorption of Structures and Materials.

[14] Masters I.G., Evans K.E. (1996). Models for the elastic deformation of honeycombs. Compos. Struct..

[15] Qi C., Jiang F., Yang S. (2021). Advanced honeycomb designs for improving mechanical properties: A review. Compos. B Eng..

[16] Hedayati R., Sadighi M., Mohammadi Aghdam M., Zadpoor A. (2016). Mechanical Properties of Additively Manufactured Thick Honeycombs. Materials.

[17] Ulkir O., Ersoy S. (2025). Hybrid Experimental–Machine Learning Study on the Mechanical Behavior of Polymer Composite Structures Fabricated via FDM. Polymers.

[18] Wei K., Yang Q., Yang X., Tao Y., Xie H., Qu Z., Fang D. (2020). Mechanical analysis and modeling of metallic lattice sandwich additively fabricated by selective laser melting. Thin-Walled Struct..

[19] Zhang Y., Lin Y., Li Y., Li X. (2021). 3D printed self-similar AlSi10Mg alloy hierarchical honeycomb architectures under in-plane large deformation. Thin-Walled Struct..

[20] Zhang X., An C., Shen Z., Wu H., Yang W., Bai J. (2020). Dynamic crushing responses of bio-inspired re-entrant auxetic honeycombs under in-plane impact loading. Mater. Today Commun..

[21] Li S., Liu Z., Shim V.P.W., Guo Y., Sun Z., Li X., Wang Z. (2020). In-plane compression of 3D-printed self-similar hierarchical honeycombs—Static and dynamic analysis. Thin-Walled Struct..

[22] Hu D., Wang Y., Song B., Dang L., Zhang Z. (2019). Energy-absorption characteristics of a bionic honeycomb tubular nested structure inspired by bamboo under axial crushing. Compos. B Eng..

[23] Jiang H., Le Barbenchon L., Bednarcyk B.A., Scarpa F., Chen Y. (2020). Bioinspired multilayered cellular composites with enhanced energy absorption and shape recovery. Addit. Manuf..

[24] Zhang J., Karagiozova D., You Z., Chen Y., Lu G. (2019). Quasi-static large deformation compressive behaviour of origami-based metamaterials. Int. J. Mech. Sci..

[25] Niknam H., Akbarzadeh A.H. (2020). Graded lattice structures: Simultaneous enhancement in stiffness and energy absorption. Mater. Des..

[26] Teuschl A.H., Neutsch L., Monforte X., Rünzler D., van Griensven M., Gabor F., Redl H. (2014). Enhanced cell adhesion on silk fibroin via lectin surface modification. Acta Biomater..

[27] Zhang W., Yin S., Yu T.X., Xu J. (2019). Crushing resistance and energy absorption of pomelo peel inspired hierarchical honeycomb. Int. J. Impact Eng..

[28] Bustihan A., Botiz I., Branco R., Martins R.F. (2025). Enhancing Mechanical Energy Absorption of Honeycomb and Triply Periodic Minimal Surface Lattice Structures Produced by Fused Deposition Modelling in Reusable Polymers. Polymers.

[29] Bodaghi M., Serjouei A., Zolfagharian A., Fotouhi M., Rahman H., Durand D. (2020). Reversible energy absorbing meta-sandwiches by FDM 4D printing. Int. J. Mech. Sci..

[30] Ganilova O.A., Low J.J. (2018). Application of smart honeycomb structures for automotive passive safety. Proc. Inst. Mech. Eng. Part D J. Automob. Eng..

[31] Khan N., Riccio A. (2024). A systematic review of design for additive manufacturing of aerospace lattice structures: Current trends and future directions. Prog. Aerosp. Sci..

[32] Wu Y., Sun L., Yang P., Fang J., Li W. (2021). Energy absorption of additively manufactured functionally bi-graded thickness honeycombs subjected to axial loads. Thin-Walled Struct..

[33] Barletta M., Gisario A., Mehrpouya M. (2021). 4D printing of shape memory polylactic acid (PLA) components: Investigating the role of the operational parameters in fused deposition modelling (FDM). J. Manuf. Process..

[34] Isaac C.W., Sokołowski A., Duddeck F., Adamiak M., Pakieła W., Aremu A. (2023). Mechanical characterisation and crashworthiness performance of additively manufactured polymer-based honeycomb structures under in-plane quasi-static loading. Virtual Phys. Prototyp..

[35] Habib F.N., Iovenitti P., Masood S.H., Nikzad M. (2017). In-plane energy absorption evaluation of 3D printed polymeric honeycombs. Virtual Phys. Prototyp..

[36] Fu X., Zhang X., Huang Z. (2021). Axial crushing of Nylon and Al/Nylon hybrid tubes by FDM 3D printing. Compos. Struct..

[37] Miralbes R., Ranz D., Pascual F.J., Zouzias D., Maza M. (2022). Characterization of additively manufactured triply periodic minimal surface structures under compressive loading. Mech. Adv. Mater. Struct..

[38] Bates S.R.G., Farrow I.R., Trask R.S. (2016). 3D printed polyurethane honeycombs for repeated tailored energy absorption. Mater. Des..

[39] Isaac C.W., Duddeck F. (2022). Current trends in additively manufactured (3D printed) energy absorbing structures for crashworthiness application—A review. Virtual Phys. Prototyp..

[40] Yuan S., Chua C.K., Zhou K. (2019). 3D-Printed Mechanical Metamaterials with High Energy Absorption. Adv. Mater. Technol..

[41] Taheri Andani M., Saedi S., Turabi A.S., Karamooz M.R., Haberland C., Karaca H.E., Elahinia M. (2017). Mechanical and shape memory properties of porous Ni 50.1 Ti 49.9 alloys manufactured by selective laser melting. J. Mech. Behav. Biomed. Mater..

[42] Kreide C., Koricho E., Kardel K. (2024). Energy absorption of 3D printed multi-material elastic lattice structures. Prog. Addit. Manuf..

[43] Lubombo C., Huneault M.A. (2018). Effect of infill patterns on the mechanical performance of lightweight 3D-printed cellular PLA parts. Mater. Today Commun..

[44] Mohamed O.A., Masood S.H., Bhowmik J.L. (2015). Optimization of fused deposition modeling process parameters: A review of current research and future prospects. Adv. Manuf..

[45] Frick A., Rochman A. (2004). Characterization of TPU-elastomers by thermal analysis (DSC). Polym. Test..

[46] Technical Data Sheet for 3D Printing FILAFLEX 70A Filament. https://drive.google.com/drive/u/1/folders/1b1YqUSY3u4PbGNSWM9uAjZ0HrjZGHYKv.

[47] Technical Data Sheet for 3D Printing Ultimaker TPU 95A Filament. n.d. https://makerbot.my.salesforce.com/sfc/p/#j0000000HOnW/a/5b000004UZS8/BFu9ADWYgMCfMUMoKa7e09bIQwrMG3AesDny5xh.myY.

[48] Technical Data Sheet for NinjaFlex^®^ 3D Printing Filament. n.d. https://ninjatek.com/wp-content/uploads/NinjaFlex-TDS.pdf.

[49] Helou M., Kara S. (2018). Design, analysis and manufacturing of lattice structures: An overview. Int. J. Comput. Integr. Manuf..

[50] Lin K., Gu D., Hu K., Yang J., Wang H., Yuan L., Shi X., Meng L. (2021). Laser powder bed fusion of bio-inspired honeycomb structures: Effect of twist angle on compressive behaviors. Thin-Walled Struct..

[51] (2019). Plastics—Determination of Tensile Properties—Part 1: General Principles.

[52] (2010). Standards Test Method for Tensile Propeties of Plastics: Annual Book of ASTM Standards.

[53] Kader M.A., Hazell P.J., Brown A.D., Tahtali M., Ahmed S., Escobedo J.P., Saadatfar M. (2020). Novel design of closed-cell foam structures for property enhancement. Addit. Manuf..

[54] Morales U., Esnaola A., Iragi M., Aretxabaleta L., Aurrekoetxea J. (2021). The effect of cross-section geometry on crushing behaviour of 3D printed continuous carbon fibre reinforced polyamide profiles. Compos. Struct..

[55] Wu X., Su Y., Shi J. (2020). In-plane impact resistance enhancement with a graded cell-wall angle design for auxetic metamaterials. Compos. Struct..

[56] Isaac C.W. (2020). Crushing response of circular thin-walled tube with non-propagating crack subjected to dynamic oblique impact loading. Int. J. Prot. Struct..

[57] Mohsenizadeh M., Gasbarri F., Munther M., Beheshti A., Davami K. (2018). Additively-manufactured lightweight Metamaterials for energy absorption. Mater. Des..

[58] Liu Y., Schaedler T.A., Chen X. (2014). Dynamic energy absorption characteristics of hollow microlattice structures. Mech. Mater..

[59] de Sousa R.A., Gonçalves D., Coelho R., Teixeira-Dias F. (2012). Assessing the effectiveness of a natural cellular material used as safety padding material in motorcycle helmets. Simulation.

