# Supplementary material for: Reusable 3D-Printed Thermoplastic Polyurethane Honeycombs for Mechanical Energy Absorption
Source: Polymers (Basel). 2025 Nov 16;17(22):3035. doi: 10.3390/polym17223035 (PMC12656569; doi:10.3390/polym17223035)
Supplement: Supplementary file 1 [file polymers-17-03035-s001.zip › polymers-3949286-supplementary.pdf]

Supplemental Information for:

Reusable 3D-Printed Thermoplastic Polyurethane Honeycombs for Mechanical Energy Absorption

Alin Bustihan[1], Razvan Hirian[1], Ioan Botiz[1,2]*

[1]Department of Physics of Condensed Matter and Advanced Technologies, Faculty of Physics, Babeș-Bolyai University, 400084 Cluj-Napoca, Romania;

[2]Interdisciplinary Research Institute on Bio-Nano-Sciences, Babeș-Bolyai University, 400271, Cluj-Napoca, Romania;

**email: ioan.botiz@ubbcluj.ro.*

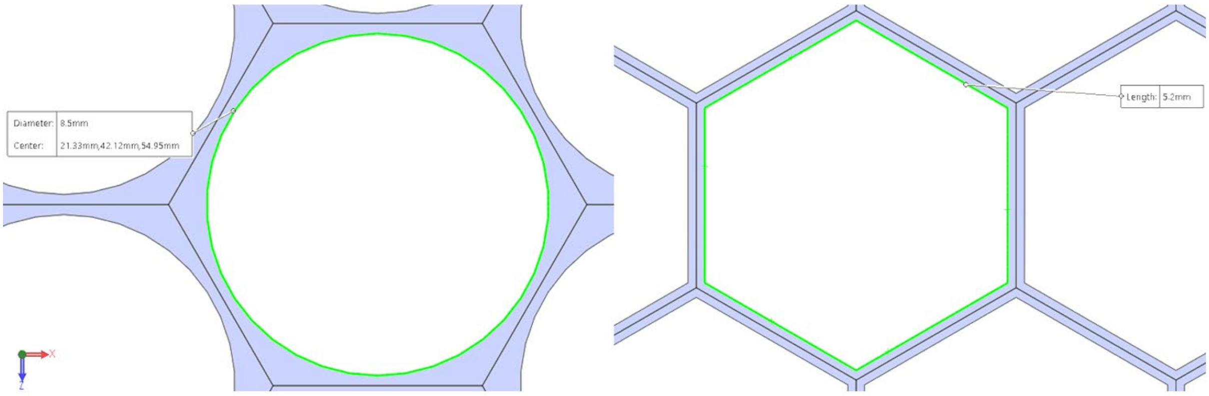

a b

Figure S1. The basic cell of the honeycomb, circle (a) and hexagon (b) structures.

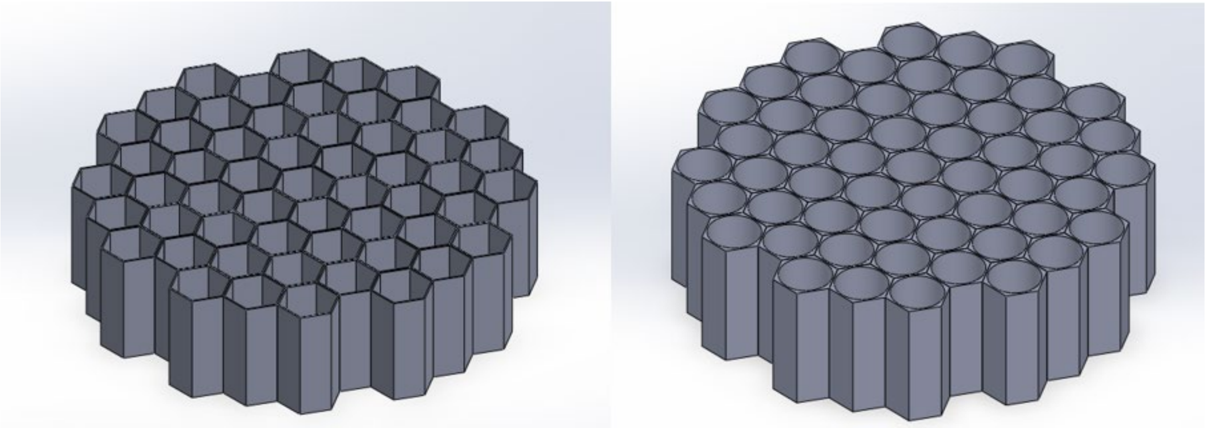

Figure S2. Honeycomb absorption structure with hexagonal holes (left) and circular holes (right).

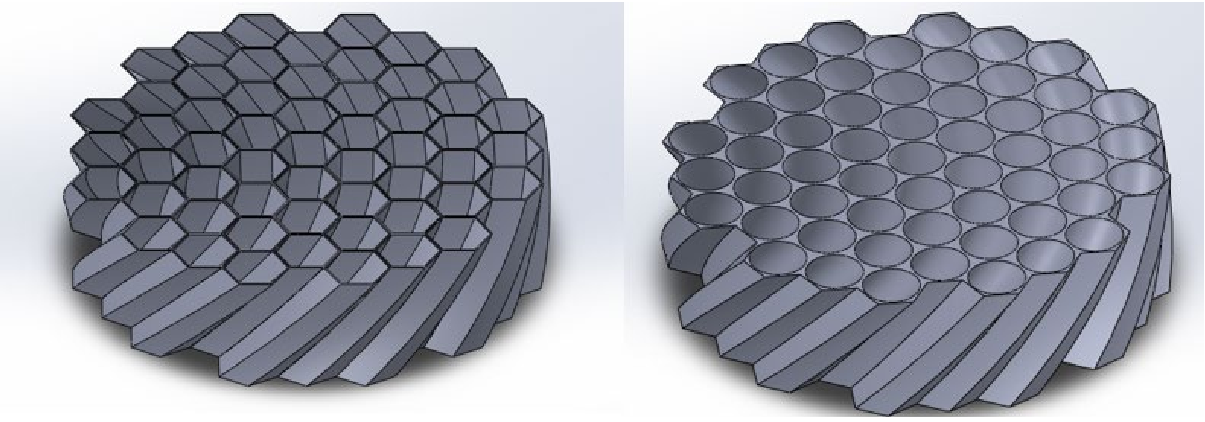

Figure S3. Honeycomb absorption structure with hexagonal holes (left) and circular holes (right), twisted with an angle of 30°.

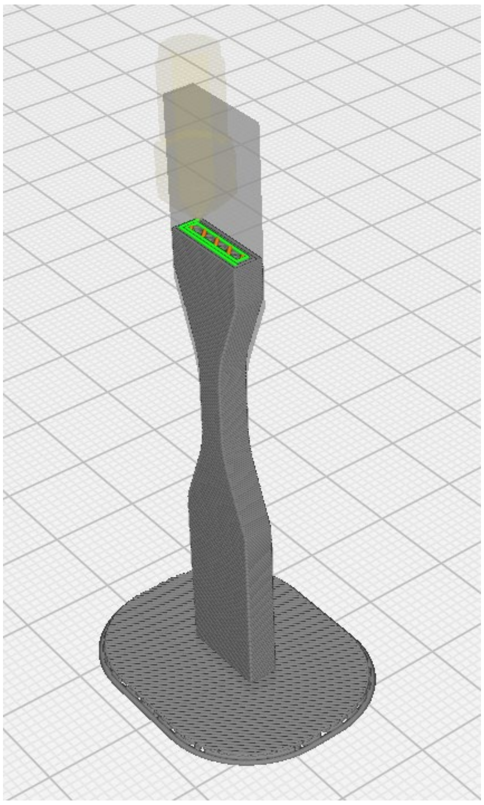

Figure S4. The printing method of standard ASTM-D628-10 Type V samples, so they can be used for layer adhesion testing.

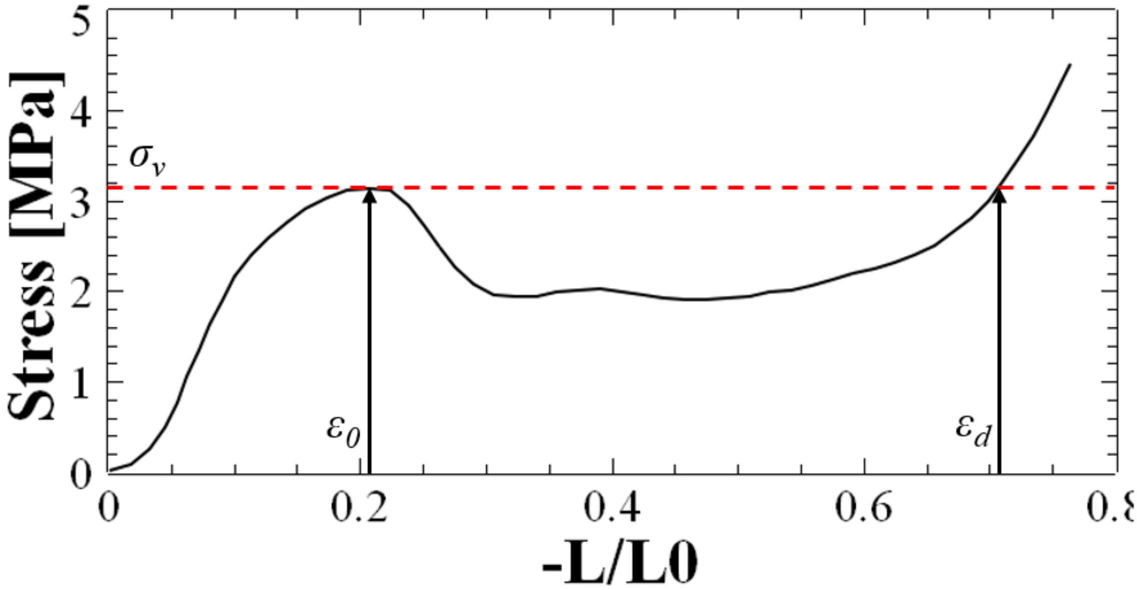

Figure S5. Absorption curve of a hexagonal holes TPU 95A structure, used to visualize the parameters σ_v, ε_d and ε_0.

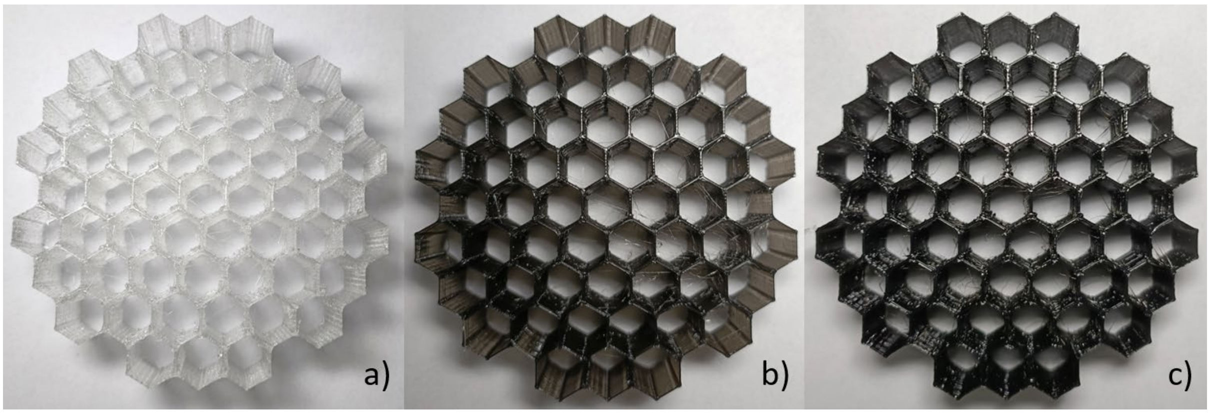

Figure S6. Honeycomb-type energy-absorbing structure with hexagonal holes internal configuration. The structures were 3D printed using: a) TPU 70A, b) TPU 85A and c) TPU 95A.

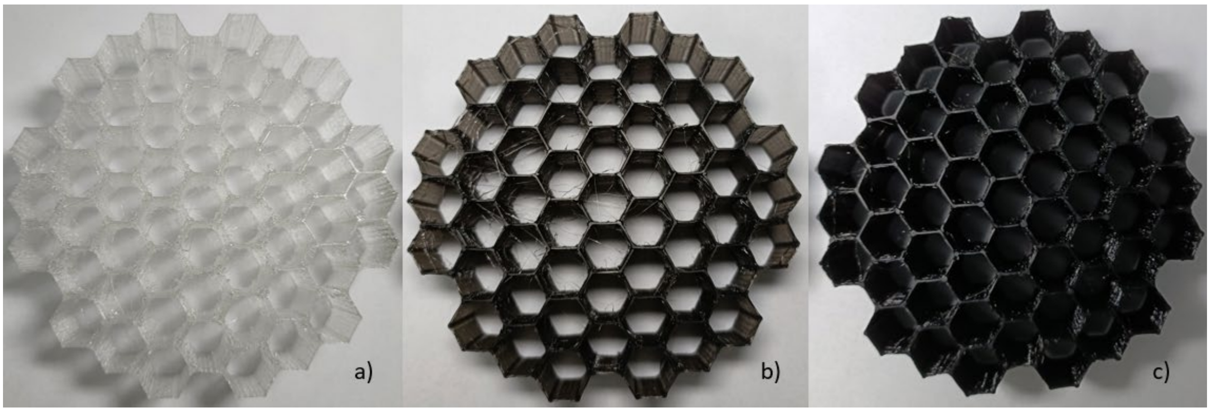

Figure S7. Honeycomb-type energy-absorbing structure with hexagonal holes internal configuration after undergoing a compression test involving three successive compressions. The structures were 3D printed using: a) TPU 70A, b) TPU 85A and c) TPU 95A.

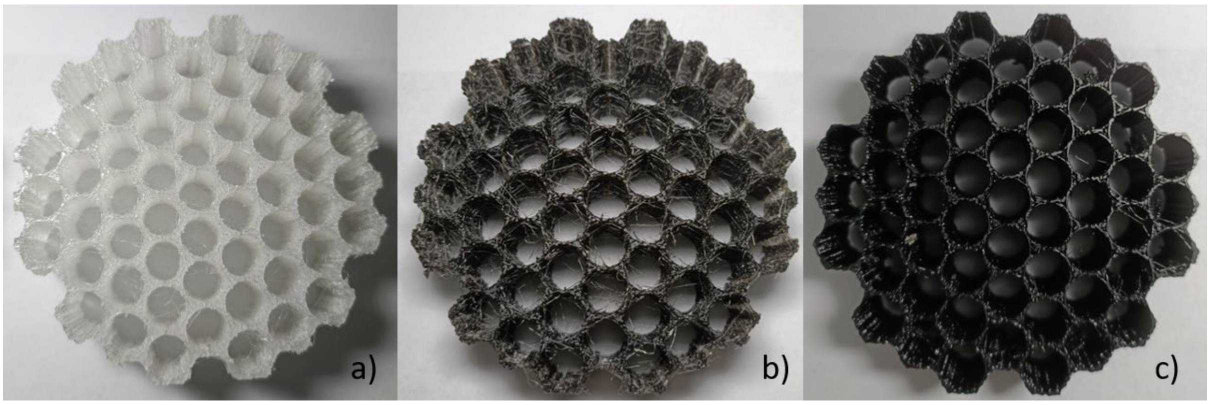

Figure S8. Honeycomb-type energy-absorbing structure with circle holes internal configuration. The structures were 3D printed using: a) TPU 70A, b) TPU 85A and c) TPU 95A.

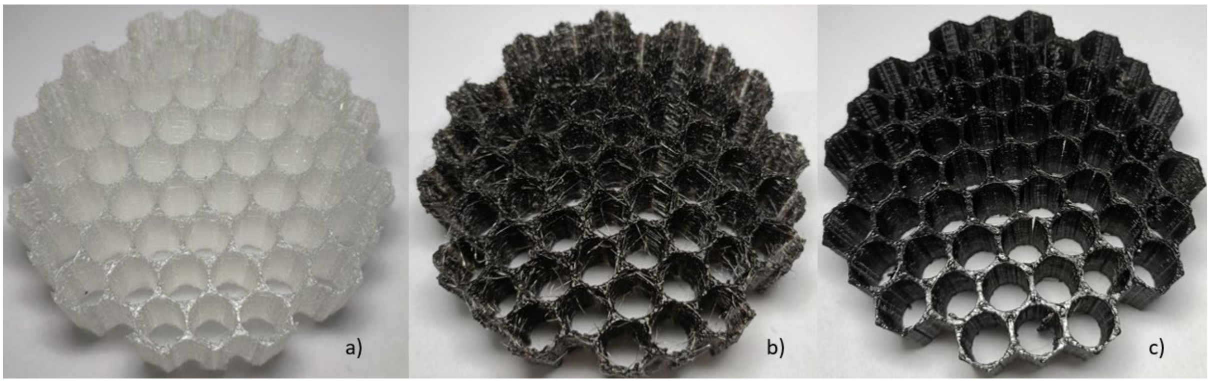

Figure S9. Honeycomb-type energy-absorbing structure with circle holes internal configuration after undergoing a compression test involving three successive compressions. The structures were 3D printed using: a) TPU 70A, b) TPU 85A and c) TPU 95A.

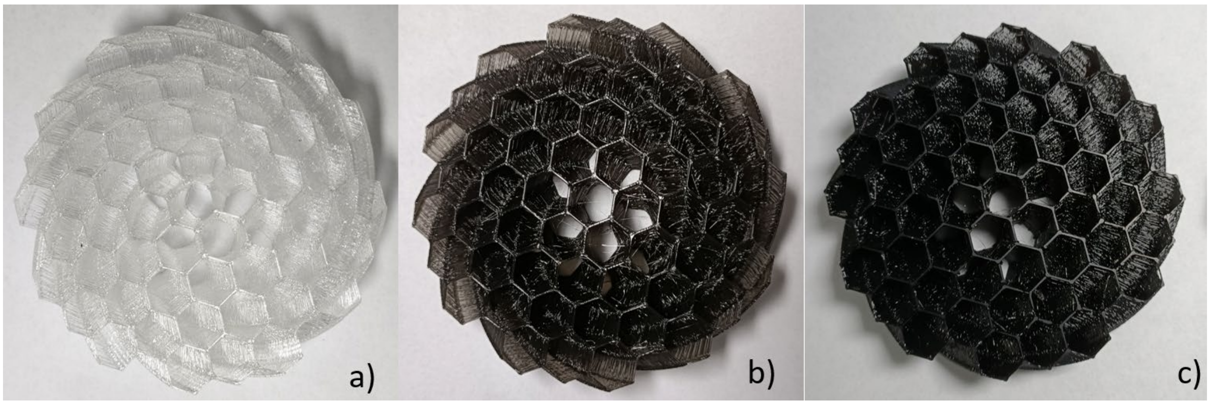

Figure S10. Honeycomb-type energy-absorbing structure with hexagonal holes, featuring the top plane twisted with an angle of 30°. The structures were 3D printed using: a) TPU 70A, b) TPU 85A and c) TPU 95A.

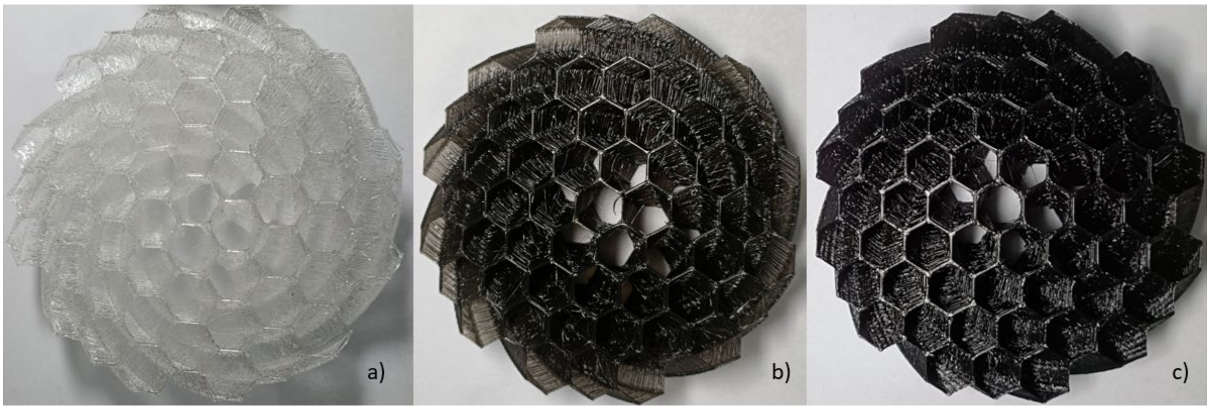

Figure S11. Honeycomb-type energy-absorbing structure with hexagonal holes, featuring the top plane twisted with an angle of 30°, after undergoing a compression test involving three successive compressions. The structures were 3D printed using: a) TPU 70A, b) TPU 85A and c) TPU 95A.

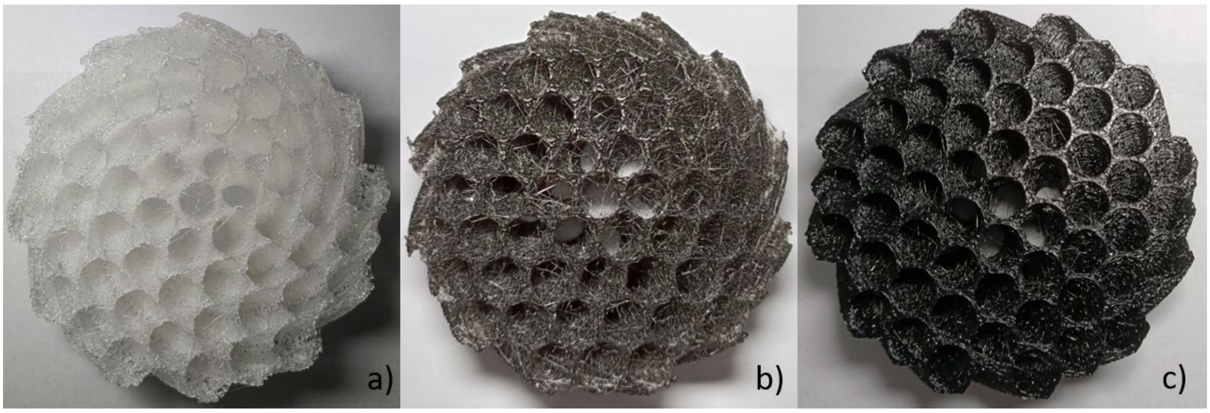

Figure S12. Honeycomb-type energy-absorbing structure with circular holes, featuring the top plane twisted with an angle of 30°. The structures were 3D printed using: a) TPU 70A, b) TPU 85A and c) TPU 95A.

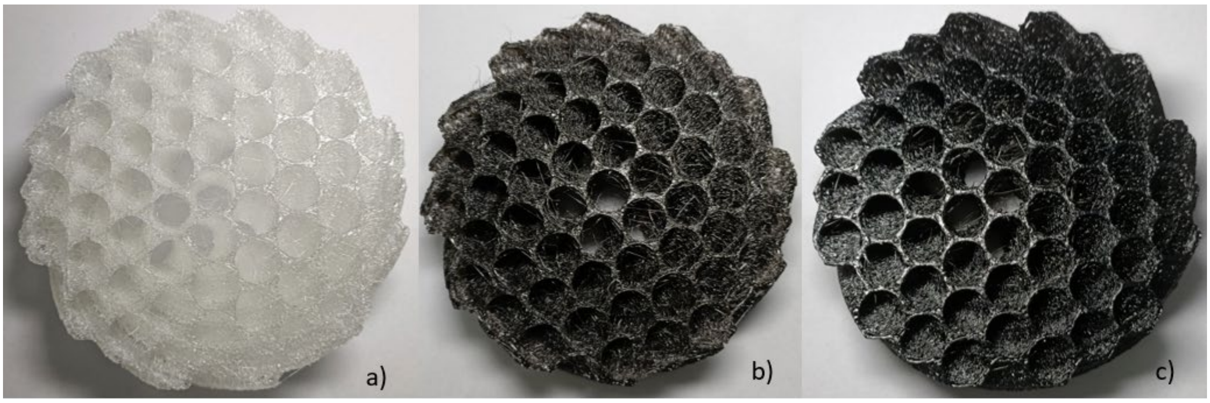

Figure S13. Honeycomb-type energy-absorbing structure with circular holes, featuring the top plane twisted with an angle of 30°, after undergoing a compression test involving three successive compressions. The structures were 3D printed using: a) TPU 70A, b) TPU 85A and c) TPU 95A.
